# Flexible Supercapacitors Based on Stretchable Conducting Polymer Electrodes

**DOI:** 10.3390/polym15081856

**Published:** 2023-04-12

**Authors:** Wen Wang, Jie Cao, Jiawen Yu, Fajuan Tian, Xiaoyu Luo, Yiting Hao, Jiyan Huang, Fucheng Wang, Weiqiang Zhou, Jingkun Xu, Ximei Liu, Hanjun Yang

**Affiliations:** 1 Jiangxi Key Laboratory of Flexible Electronics, Flexible Electronics Innovation Institute, Jiangxi Science & Technology Normal University, Nanchang 330013, China; 2 School of Chemistry and Chemical Engineering, Jiangxi Science & Technology Normal University, Nanchang 330013, China; 3 School of Pharmacy, Jiangxi Science & Technology Normal University, Nanchang 330013, China; 4 School of Chemistry and Molecular Engineering, Qingdao University of Science and Technology, Qingdao 266042, China

**Keywords:** flexibility, conducting polymer, supercapacitors

## Abstract

Supercapacitors are widely used in various fields due to their high power density, fast charging and discharging speeds, and long service life. However, with the increasing demand for flexible electronics, integrated supercapacitors in devices are also facing more challenges, such as extensibility, bending stability, and operability. Despite many reports on stretchable supercapacitors, challenges still exist in their preparation process, which involves multiple steps. Therefore, we prepared stretchable conducting polymer electrodes by depositing thiophene and 3-methylthiophene on patterned 304 stainless steel (SS 304) through electropolymerization. The cycling stability of the prepared stretchable electrodes could be further improved by protecting them with poly(vinyl alcohol)/sulfuric acid (PVA/H_2_SO_4_) gel electrolyte. Specifically, the mechanical stability of the polythiophene (PTh) electrode was improved by 2.5%, and the stability of the poly(3-methylthiophene (P3MeT) electrode was improved by 7.0%. As a result, the assembled flexible supercapacitors maintained 93% of their stability even after 10,000 cycles of strain at 100%, which indicates potential applications in flexible electronics.

## 1. Introduction

With the increasing demand for clean and sustainable energy, the development of energy storage devices has become increasingly important. Among the various energy storage options available, supercapacitors stand out, with their exceptional advantages such as high power density, fast charging and discharging, and long cycling lifetime. These features make supercapacitors a promising technology for a wide range of applications, including portable electronics, electric vehicles, and renewable energy systems [1,2,3,4,5,6]. Recent advancements in the field of energy storage have primarily concentrated on enhancing the energy density of electrodes and increasing the speed of charging [7,8,9,10,11,12,13]. Stretchable supercapacitors where flexibility and stretchability functions are needed, have been studied in order to meet demand for the rapid progress in modern portable electronic devices and wearable smart consumer electronics [14,15,16,17]. In particular, to satisfy the requirement for stretchable supercapacitors, the integration of electrode materials and substrate is extensively studied [18,19,20,21,22]. However, the reported supercapacitors typically suffer from a complex processing method [23,24,25,26]. For example, through the ink-based blending of thermally annealed graphene oxide (GO) with ethyl cellulose (EC) and terpineol, which was then printed on a polyimide (PI) substrate by 3D printing, Y. Wang et al. developed a flexible supercapacitor for application in portable microelectronics systems [27]. Despite recent advances in reducing the procedure by combining electrode materials with electrolytes, flexible supercapacitors still suffer from poor electrochemical stability due to the wear of electrode material during the tensile deformation process [28,29,30]. In addition, flexible supercapacitors are often prone to inefficient charge transfer and energy storage for the use of additives that provide stretching properties with decreased conductivity of the electrode material [31,32,33]. However, the choice of electrode material is a key factor in determining the performance and durability of the flexible supercapacitor, as it directly affects the capacitance, charge/discharge rate, and cycling stability. Therefore, extensive research has been conducted to identify novel electrode materials that exhibit desirable electrochemical properties and can be fabricated using scalable techniques.

Conducting polymers have gained increasing attention as electrode materials for making flexible supercapacitors due to their high conductivity, good processability, and electrochemical stability [34,35,36]. However, conducting polymers also have some limitations when used as electrode materials in supercapacitors. One of the main drawbacks is their relatively low stability over repeated cycling, which can lead to a decrease in performance over time [37,38]. Furthermore, conducting polymers can be mechanically unstable, especially when subjected to large strains, which can cause cracking and delamination [39]. Alternatively, researchers are exploring the use of hybrid materials that combine conducting polymers with other materials, such as carbon nanotubes or metal oxides, to enhance the mechanical and electrochemical stability of the electrodes. Currently, this method is still limited by complex preparation methods and high costs.

Here, we propose a simple method for preparing flexible supercapacitors using conducting polymers and patterned current collectors, which exhibit good electrochemical stability even after longtime cyclic stretching. Firstly, one-step electropolymerization was developed to generate flexible electrodes of conducting polymers by depositing polythiophene (PTh) and poly(3-methylthiophene) (P3MeT) directly onto patterned 304 stainless steel (SS 304) current collectors, respectively. By controlling the collectors’ patterns, the current density can be more evenly distributed across the electrode [40], which can reduce the risk of electrode degradation and improve the lifetime of the supercapacitor [41,42]. Secondly, we utilized a poly(vinyl alcohol)/sulfuric acid (PVA/H_2_SO_4_) hydrogel as a gel electrolyte to provide further protection to the electrode materials [43,44], followed by adhering PDMS films on both sides to enhance stability. We then demonstrated that the patterned SS 304 current collectors can simultaneously display high stretchability (250%) and plastic deformation (<3.5%). The flexible supercapacitor also maintained 93% of its original capacity after 10,000 stretching cycles. To demonstrate the enhanced properties of flexible supercapacitors, we depict their application as energy storage devices. The practical applications of our flexible supercapacitor based on stretchable conducting polymer electrodes show the enormous potential in next-generation portable electronic devices and flexible electronics.

## 2. Materials and Methods

### 2.1. Materials

Thiophene (98%; Aladdin, Shanghai, China), 3-methylthiophene (3MeT, 98%; Aladdin, Shanghai, China), anhydrous lithium perchlorate (LiClO_4_, 98%; Xiya Reagent Research center, Shandong, China), boron trifluoride diethyl etherate (BFEE, 48%; J&K Chemicals, Beijing, China), and acetonitrile (ACN, 99.90%; J&K Chemicals, Beijing, China) were used directly for the electropolymerization. Other reagents such as sulfuric acid (H_2_SO_4_, 98%; Xi-Long Chemical, Guangdong, China), polyvinyl alcohol (PVA, Mw = 146,000–186,000; Sigma-Aldrich, Shanghai, China), polydimethylsiloxane (PDMS, 184 silicone elastomer; Dow Corning, Shanghai, China), ethanol (95%; Aladdin, Shanghai, China), and hydrochloric acid (HCl, 37%; Beijing Chemical Reagents, China) were utilized without any further purification. Other chemicals were analytical-grade and used as received.

### 2.2. Fabrication of Flexible Supercapacitor

*Fabrication of stretchable current collector.* The development of materials with stretchable properties has become increasingly important in recent years due to the growing demand for flexible electronic devices and wearable technologies. However, achieving stretchability in traditionally rigid materials such as metals can be a challenging task. One approach to achieving stretchability in metals is through the use of structural design. By incorporating serpentine structures into the material, it is possible to achieve large ductility and stretchability while maintaining its overall structural integrity. The 304 stainless steel (SS 304; 0.3 mm diameter) was chosen as the material of current collector due to its favorable mechanical properties. To design a stretchable current collector, the SS 304 was optimized by varying its basic parameters such as width (W), length (L), and adjacent spacing (A). These parameters were carefully selected to achieve the desired mechanical properties, such as high ductility and stretchability, while maintaining a stable resistance. By comparing different ratios of these parameters in experiments, an optimized current collector was designed with a length of 6 cm, an adjacent spacing of 0.2 cm, and a maximum height of 5 cm.

*Preparation of electrodes*. Cyclic voltammetry (CV) polymerization is an electrochemical method used to form conducting polymers on the SS 304 current collector surface. During the CV process, a potential range is applied to the electrode in the presence of monomer and supporting electrolyte. This leads to the formation of a thin film of the polymer on the electrode surface. In this study, P3MeT and PTh electrodes were synthesized using the electrochemical method, specifically through potentiostatic technique on an electrochemical workstation (CHI660E-A1385; CH Instruments Ins, Austin, TX, USA) on SS 304 current collector. To ensure the optimal performance of the electrodes, the SS 304 current collector was thoroughly cleaned prior to the electrodeposition process. This involved washing the current collector with ethanol and deionized water in sequence, followed by drying under nitrogen flow. This process was crucial to remove impurities that may affect the surface wettability of the SS 304 current collector. Once the current collector was cleaned and dried, it was immersed in 50 mL of can electrodeposition solution [3MeT (480 µL, 5 mmol), LiClO_4_ (530 mg, 5 mmol), and BFEE (2.5 mL, 10 mmol)] at room temperature. The P3MeT polymer was then deposited on the SS 304 current collector using cyclic voltammetry (CV) polymerization. The deposition potential range used for P3MeT electrodeposition was from −0.2 to 1.25 V. Similarly, the PTh electrodes were also prepared using the same method, but with a deposition potential range from −0.6 to 1.4 V.

*Preparation of PVA/H_2_SO_4_ gel electrolyte*. The electrolyte is a critical component of energy storage devices, and the selection of an appropriate electrolyte can greatly impact the overall performance of the device. The PVA/H_2_SO_4_ gel electrolyte was prepared using the physical crosslinking method. The preparation of the PVA/H_2_SO_4_ solution involved dissolving 5 g of PVA powders and 5 mL of H_2_SO_4_ into 45 mL of deionized water (DI) at 90 °C while constantly stirring. The solution was kept at this temperature until a homogeneous, transparent precursor solution was obtained. To form the PVA/H_2_SO_4_ gel electrolyte, the precursor solution was subjected to three cycles of freezing and thawing. During this process, the precursor solution was frozen at a temperature of −20 °C for 8 h and then thawed at 25 °C for 3 h. This freeze–thaw process led to the formation of a gel electrolyte with a three-dimensional network structure, which was stabilized by physical crosslinking between the PVA chains.

*Assembly of flexible supercapacitor*. Firstly, the P3MeT electrode was immersed in a square mold containing the PVA/H_2_SO_4_ precursor solution. The mold was then subjected to three cycles of freeze–thaw, where the temperature was lowered to −20 °C for 8 h, and then raised to 25 °C for 3 h, in order to form a gel electrolyte. Next, the PTh electrode was placed on top of the PVA/H_2_SO_4_ gel electrolyte in the mold, and the precursor solution was added to fill the mold. The entire assembly was then subjected to successive multiple freeze–thaw cycles to form a gel electrolyte with two electrodes. To enhance the stability of the flexible supercapacitor and make it suitable for portable electronic devices, it was encapsulated with PDMS, which acts as a protective layer. Once the encapsulation process is complete, the flexible supercapacitor is ready for use.

### 2.3. Characterizations

*Electrochemical measurements.* The electrochemical properties were studied by the electrochemical analyzer (CHI660E-A1385; CH Instruments Ins, Austin, TX, USA) in 1 M H_2_SO_4_ aqueous electrolyte with the platinum sheet electrode as the counter electrode and the saturated Ag/AgCl wire as the reference electrode (room temperature, 25 °C). The electrochemical characterizations used CV and galvanostatic charge–discharge (GCD).

Areal capacity (*C_a_*) values of varying samples are calculated according to the following Equation:

$$C=\frac{I\Delta t}{\Delta E}$$

$$C_{a}=\frac{C}{S}=\frac{I\Delta t}{S\Delta E}$$

where *C* (mF) and *I* (A) correspond to the total capacitance and discharge current. *Δt* (s) is the time of discharge process; *ΔE* (V) is the potential window during the measurement; *S* (cm^2^) is the effective area of the electrodes.

*Mechanical characterization*. Mechanical properties of the stretchable current collector, electrodes, and flexible supercapacitor were performed by a universal testing machine (DWD-010 with 100 N load cell, Changchunkexin Precision Instrument) equipped at a rate of 50 mm/min for the mechanical test (room temperature 25 °C).

*Fourier-transform infrared (FTIR) spectrometer*. The FTIR spectra of thiophene, 3-methylthiophene, PTh, and P3MeT were analyzed using FTIR spectrometer (Spectrum Two, Perkin-Elmer) in the wavelength range of 4500–500 cm^−1^, with a spectroscopy resolution of approximately 4 cm^−1^. The spectra were obtained by averaging 32 scans at room temperature (25 °C).

*Resistance measurement*. The four-point probe measurement is a widely used technique for measuring resistance with high accuracy. In this study, the resistance of the stretchable SS 304 current collector was measured by a standard four-point probe (Keithley 2700 digital multimeter, Keithley, Beaverton, OG, USA) at 25 °C. To measure the resistance of the current collector under different stretching cycles, copper electrodes were adhered to the surface of the current collector. This allowed for the calculation of the resistance of the current collector at different stretching cycles.

## 3. Results

### 3.1. Design of Flexible Supercapacitor

**Figure 1:**
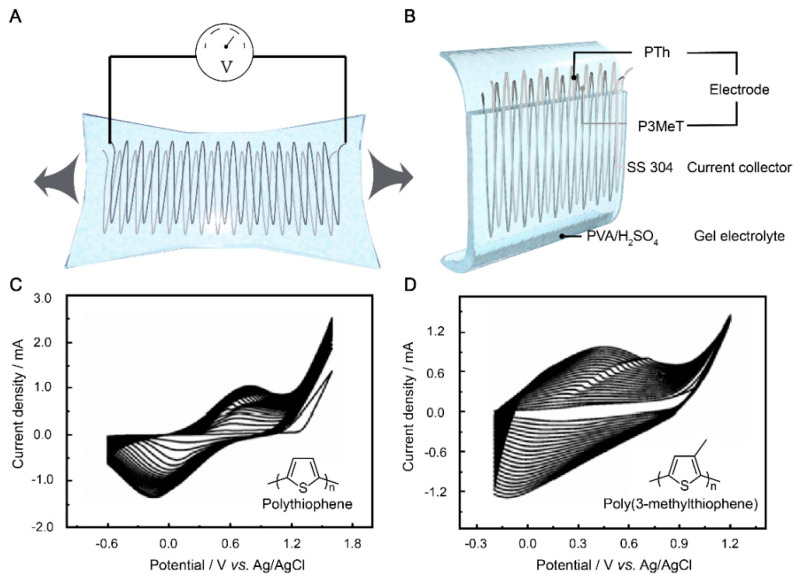
(**A**) Design of flexible supercapacitor. (**B**) Schematic diagram of the flexible supercapacitor. The continuous CV curves of PTh (**C**) and P3MeT (**D**) electrodeposited in 0.2 M BFEE/ACN solution, respectively.

Owing to their superior electrical conductivity, processability, and environmental properties, conducting polymers have emerged as promising candidate electrode materials for the fabrication of flexible supercapacitors [45,46,47]. However, conducting polymers suffer from poor stability when used as electroactive materials due to their shape changes. Based on the requirement of a flexible supercapacitor, we designed SS 304 wire as a current collector and PVA/H_2_SO_4_ as a gel electrolyte to protect a conducting polymer electrode consisting of P3MeT and PTh. The use of PVA as a matrix material for the gel electrolyte is advantageous due to its high water retention ability, low toxicity, and excellent biocompatibility. Additionally, H_2_SO_4_ was added to the PVA matrix to enhance its ionic conductivity. H_2_SO_4_ dissociates in water to form H^+^ and SO_4_^2-^ ions, which can act as charge carriers in the electrolyte. The resulting PVA/H_2_SO_4_ gel electrolyte exhibited excellent mechanical stability, good ionic conductivity, and high compatibility with the electrode materials. First, thiophene and 3-methylthiophene were electrochemically polymerized on the pre-engineered stretchable SS 304 wire, respectively. The electrodes were then placed in a precursor solution of PVA/ H_2_SO_4_ and then condensed into a solid gel electrolyte to further prevent PTh and P3MeT from falling off the SS 304 wire during the stretching cycles (Figure 1B).

We designed serpentine wires with different parameters before assembling a flexible supercapacitor, which ensured good tensile properties during the synthesis of the electrodes through the electrochemical method (detailed information is available in the experimental data and rate optimization). The P3MeT and PTh electrodes were prepared by electrochemical polymerization at a scan rate of 100 mV s^−1^ and −0.2–1.25 V and −0.6–1.4 V, respectively (Figure 1C,D). As a result, it can be seen in the continuous CV curve that as the number of CV scan turns increases, the current density increases. It also indicates that the number of electrode materials on the surface of the SS 304 wire increases. However, two conductive polymers, thiophene and 3-methylthiophene, were electropolymerized into different electrodes at varying voltages. The current density of the PTh electrode per cycle was significantly higher than that of P3MeT, which is attributed to the presence of the methyl side group on thiophene, which can further increase the steric effect [48].

### 3.2. P3MeT and PTh as Active Electrode Materials for Supercapacitor

**Figure 2:**
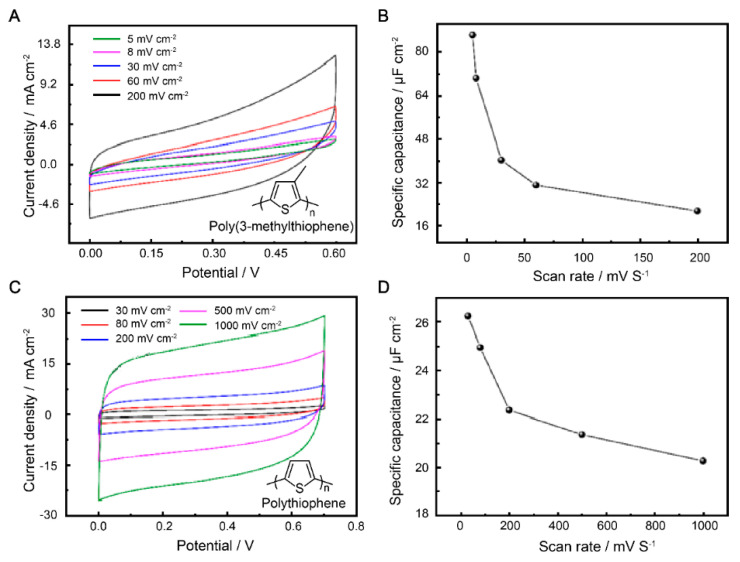
Electrochemical performance of P3MeT and PTh electrodes in 1 M H_2_SO_4_ electrolyte. (**A**) CV curves of P3MeT at scan rates of 5–200 mV cm^−2^. (**B**) Areal capacity of P3MeT at various voltages with the scan rates of 5–200 mV cm^−2^. (**C**) CV curves of PTh at scan rates of 5–200 mV cm^−2^. (**D**) Areal capacity of PTh at various voltages with scan rates of 5–200 mV cm^−2^.

The electrochemical behaviors of P3MeT and PTh were further explored in a three-electrode system. The different behaviors were correlated with electrochemical kinetics, and the two electrodes were studied by CV at various scan rates (5–200 mV s^−1^ and 30–1000 mV s^−1^). Compared to the shape of the CV curves, there was a change in peak height at low scan rates for both electrodes, accompanied by a positive shift in the oxidation peak potential and a negative shift in the reduction peak potential (Figure 2A,C). These changes are mainly attributed to the resistance of the electrode. The areas enclosed by the CV curves increased with the scan rates of all electrode samples. On the one hand, the two electrodes were found to reach the highest capacitance at 0–0.7 V, which is in line with most reports on supercapacitor electrode materials based on polythiophene conductive polymers. On the other hand, the symmetric rectangular behavior of the two different electrodes indicates an extremely rapid current response on voltage reversal.

The *C*_a_ of the electrode was obtained by the integral area of CV curves (Figure 2B,D). The P3MeT and PTh electrode materials exhibited different electrochemical reaction rates and response rates. Additionally, different scan rates can affect the electrochemical stability and durability of the capacitors. To demonstrate a consistent trend of capacitance decline during scanning, we selected various scanning rates from the numerous experimental results for evaluating the performance of the two conductive polymer electrode materials. It can be seen that the correlation between the scan rates and *C*_a_ of the electrodes is plotted in the picture. While the maximum *C*_a_ of P3MeT electrode be obtained at a scan rate of 5 mV s^−1^ with a *C*_a_ value of 87.2 µF cm^−2^, the *C*_a_ value of PTh electrode is 26.4 µF cm^−2^ at a scan rate of 30 mV s^−1^. However, the *C*_a_ values of the electrode materials decreased with the enlarged scan rate due to two reasons. Firstly, the decline in *C*_a_ was due to the fact that at low scan rates, SO_4_^2−^ has enough time to enter and exit the conducting polymer electrodes. However, at higher scan rates, the diffusion of SO_4_^2−^ does not have enough time to come into contact with the electrode. Secondly, ions contact the conducting polymer electrodes to a lesser extent at higher scan rates, leading to a decrease in the *C*_a_ value of both the P3MeT and PTh.

### 3.3. Stability of Bare Stretchable Electrode for Supercapacitor

**Figure 3:**
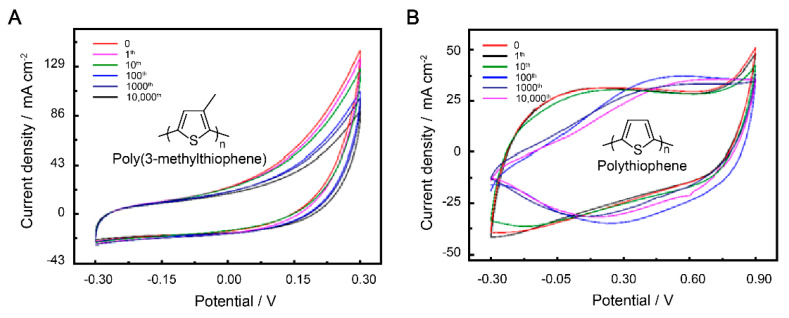
CV curves of P3MeT (**A**) and PTh (**B**) bare stretchable electrodes after 10,000 cycles of strain at 100%.

In order to assess the suitability of our patterned electrodes for potential applications, such as flexible sensors and wearable electronics, we conducted mechanical testing by measuring the CV curves and cycling performance. The mechanical flexibility of the SS 304 wire current collector was evaluated through stretching tests (Figure S1). When the width of the patterned electrodes (W) is 5 cm and the width between two adjacent SS 304 lines (A) is 0.2 cm, only a small deformation is generated, resulting in a 3% increase in initial resistance after more than 10,000 cycles of stretching at 250% (Figure S2). After subjecting the patterned current collector to mechanical testing, the electrochemical performance of the supercapacitors based on P3MeT and PTh electrodes were evaluated by measuring their CV curves. The supercapacitors were subjected to 10,000 cycles of strain at 100% to simulate real-world usage. The resulting CV curves were compared for both P3MeT and PTh electrodes, and the distinct differences observed suggest that the electrodes demonstrated stable electrochemical performance during the first 10 cycles of testing (Figure 3A,B). However, despite their stable performance during the initial cycles of testing, the electrodes did experience some swelling due to stretching and external disturbances. This swelling caused an increase in the contact distance between the electrodes and the SS 304 current collector. If this distance becomes too great, there is a potential risk of detachment, which could significantly impact the performance of the supercapacitors. Therefore, it is necessary to further protect and test the electrodes to ensure their application.

### 3.4. Stability of Electrode Materials Based on PVA/H_2_SO_4_ Gel Electrolyte Protection

**Figure 4:**
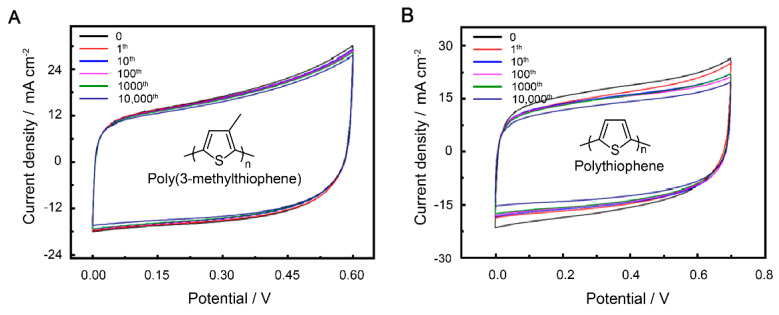
CV curves of P3MeT (**A**) and PTh (**B**) stretchable electrode after 10,000 cycles of strain at 100% with PVA/H_2_SO_4_ gel electrolyte protection.

It is notable that stability is an important parameter for supercapacitors. Therefore, to ensure that the electrode materials have excellent electrochemical stability even under stretching, we chose PVA/H_2_SO_4_ as the gel electrolyte to support the electrode [49,50]. The solid supercapacitor can charge and discharge at several voltage windows. The optimal voltage window according to the performance of P3MeT and PTh was set as 0–0.60 V (Figure 4A) and 0.70 V (Figure 4B). To prevent mechanical failure while stretching, the two electrodes were tightly embedded in a soft hydrogel environment formed by freeze–thawing the PVA/H_2_SO_4_ precursor aqueous solution for three cycles at −20 °C for 8 h and thawing at 25 °C for 3 h. The PVA/H_2_SO_4_ gel electrolyte exhibited excellent mechanical stability, preventing rupture of the electrodes while stretching. Compared to the bare electrodes, the PVA/H_2_SO_4_ gel electrolyte-protected electrodes showed better capacitance retention performance, as indicated in Table 1. The P3MeT electrode exhibited exceptional cycling stability, retaining approximately 93.0% of its areal capacitance after undergoing 10,000 cycles of strain at 100%. The intriguing results highlight the potential of using conducting polymers protected by gel electrolyte as a promising method for increasing the lifetime of supercapacitors (Figure S4). As the scan rate increased, the CV curve showed a similar quasi-rectangular shape, indicating that the electrode displayed fine capacitive behavior. Overall, the use of PVA/H_2_SO_4_ as the gel electrolyte and the embedding of the electrodes in a soft hydrogel environment significantly improved the stability and performance of the solid supercapacitor, making it a promising candidate for use in stretchable electronic devices.

### 3.5. GCD of P3MeT and PTh Electrodes

**Figure 5:**
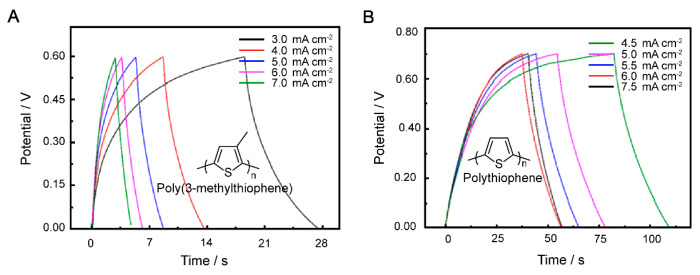
GCD curves of P3MeT (**A**) and PTh (**B**) at 3–7 mA cm^−2^ and 4.5–7.5 mA cm^−2^, respectively.

The GCD curve of the P3MeT electrodes at current densities of 3.0, 4.0, 5.0, 6.0, and 7.0 mA cm^−2^ in the potential window range from 0.00 to 0.60 V (Figure 5A), and the PTh electrodes at current densities of 4.0, 4.5, 5.0, 5.5, 6.0, and 6.5 mA cm^−2^ in the potential window range from 0.00 to 0.70 V, are demonstrated (Figure 5B). In the context of energy storage devices, the performance of the electrodes is critical for the overall device performance. The GCD curves are widely used to evaluate the electrochemical performance of electrodes. In the present study, the GCD curves of P3MeT and PTh electrodes were evaluated at various current densities and in different potential window ranges. The semisymmetric triangle shapes of the GCD curves indicate the pseudocapacitance contribution to the capacitance of the electrodes. The deviation from linearity in the potential–time relationships further confirms the presence of pseudocapacitance. The discharge curves of the electrodes also show a concave, which can be attributed to the internal resistance of the electrodes and the capacitive characteristics of the pseudocapacitive electrodes. Despite the nonlinearity of the potential–time relationships and the concave shape of the discharge curves, the GCD curves of both P3MeT and PTh electrodes show nearly symmetrical shapes under diverse charge–discharge rates. This suggests that the electrodes have perfectly reversible charge–discharge behavior, indicating good electrochemical stability. Comparing the electrochemical performance of P3MeT and PTh electrodes, it was found that P3MeT electrodes have lower internal resistance and higher pseudocapacitance. Therefore, the discharge efficiency of P3MeT electrodes is higher than that of PTh electrodes at 4.0 and 6.0 mA cm^−2^. This suggests that P3MeT electrodes have the potential to be used as high-performance electrodes for energy storage devices.

### 3.6. Electrochemical Impedance Spectroscopy of P3MeT and PTh Electrodes Based Flexible Supercapacitor

**Figure 6:**
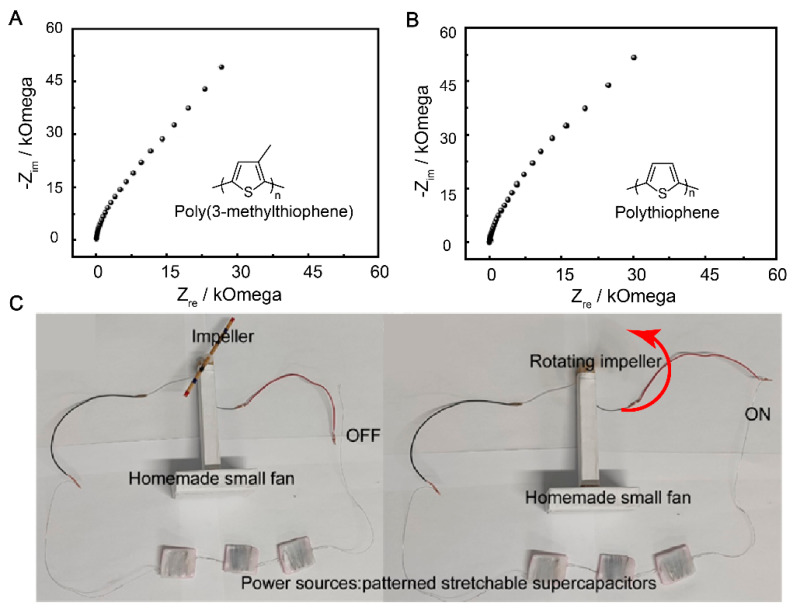
The application demo of the flexible supercapacitor. (**A**) Nyquist plots of the P3MeT electrode. (**B**) Nyquist plots of the PTh electrode. (**C**) Photograph of the flexible supercapacitor device rotating a small fan.

The use of flexible supercapacitors has been gaining popularity due to their potential applications in portable devices and other flexible and conformable applications [51]. The choice of SS 304 wire as a current collector in the fabrication of the flexible supercapacitor device is significant, as it possesses good conductivity and corrosion resistance properties. The flexible supercapacitor device was fabricated using P3MeT and PTh as the negative and positive electrodes, respectively, in a PVA/H_2_SO_4_ gel electrolyte. The Nyquist plots of the P3MeT and PTh electrodes show excellent capacitive behavior, with the EIS plot slope approaching 90° in the low-frequency region (Figure 6A,B). This indicates the promising performance of the device in terms of energy storage capacity. The potential practical application of the flexible supercapacitor device was demonstrated by connecting three devices in series to rotate a fan (Figure 6C). This emphasizes the scalability and adaptability of the device for use in various electronic applications. Overall, the use of flexible supercapacitor devices with the PVA/H_2_SO_4_ gel electrolyte, P3MeT and PTh electrodes, and SS 304 wire as a current collector provides a promising platform for the development of efficient, reliable, and flexible energy storage systems with a wide range of potential applications.

## 4. Conclusions

In conclusion, a flexible supercapacitor based on the readily available PTh and P3MeT with patterned SS 304 wires was developed. This eliminates the need for a separate step of attaching the electrode material to the current collector, streamlining the manufacturing process, and achieving a reduction in production costs. By protecting these electrodes with the PVA/H_2_SO_4_ gel electrolyte, we demonstrate their good electrochemical stability, maintaining 93% of their original capacity after 10,000 cycles of strain at 100% with an areal capacity volumetric of 87.2 µF cm^−2^. These flexible supercapacitors prepared in three simple steps exhibit excellent mechanical flexibility and high electrochemical stability during the stretching cycle. This work also suggests that combining readily available conducting polymers with commercial products is an effective strategy for designing flexible supercapacitors in an easy way.

## Figures and Tables

**Table 1:** Capacitance retention of P3MeT and PTh after different stretching cycles in bare states and gel electrolyte protection.

Electrodes	Stability					
	0	1th	10th	100th	1000th	10,000th
P3MeT	100%	97.4%	96.2%	93.3%	91.8%	86.9%
PTh	100%	98.5%	94.7%	89.2%	84.2%	83.3%
P3MeT (Gel protection)	100%	99.6%	98.1%	96.7%	95.6%	93.0%
PTh (Gel protection)	100%	94.6%	92.8%	89.9%	86.4%	85.4%

## Data Availability

The raw/processed data required to reproduce these findings cannot be shared at this time due to technical limitations. They are available upon request.

## Supplementary Materials

The following supporting information can be downloaded at: https://www.mdpi.com/article/10.3390/polym15081856/s1. Figure S1. Schematic diagram and photograph of the stretchable SS 304 current collector. Table S1. The influence of different parameters on the deformation of collector. Figure S2. The resistance and change of current collector after stretching at 250%. Figure S3. FTIR spectra of thiophene, polythio-phene, 3-methylthiophene, and poly(3-methylthiophene). Figure S4. Comparison of conducting pol-ymer electrodes protected by gel electrolyte with previously reported electrode materials under varying cyclic deformation duration.
