# Supplementary material for: Flexible Supercapacitors Based on Stretchable Conducting Polymer Electrodes
Source: Polymers (Basel). 2023 Apr 12;15(8):1856. doi: 10.3390/polym15081856 (PMC10144423; doi:10.3390/polym15081856)
Supplement: Supplementary file 1 [file polymers-15-01856-s001.zip › polymers-2292857-supplementary.pdf]

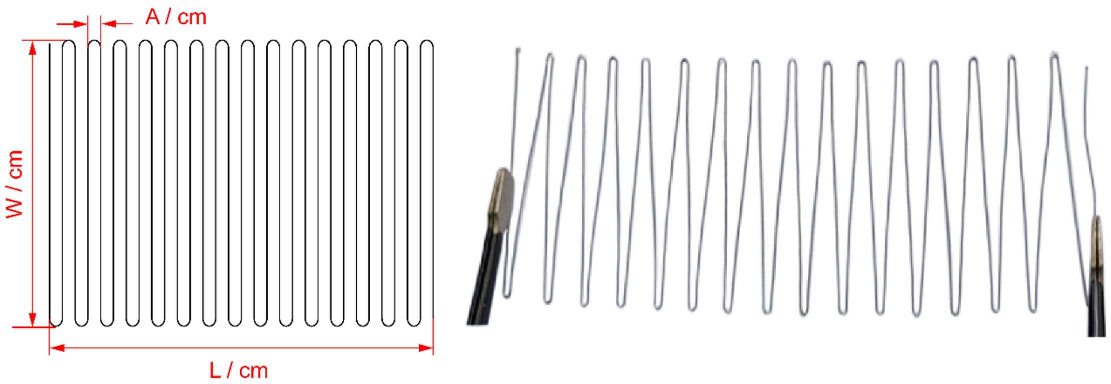

Figure S1. Schematic diagram and photograph of the stretchable SS 304 current collector.

Table S1. The influence of different parameters on the deformation of collector.

W / cm	6<br>Stretchability/ plastic deformation				
H / cm	5<br>250% / 3.5%	4<br>170% / 4.3%	3<br>120% / 3.3%	2<br>60% / 5%	(L = 0.2)
L / cm	0.6<br>90% / 3.3%	0.5<br>170% / 5%	0.4<br>190% / 10%	0.3<br>200% / 4%	(H = 5)

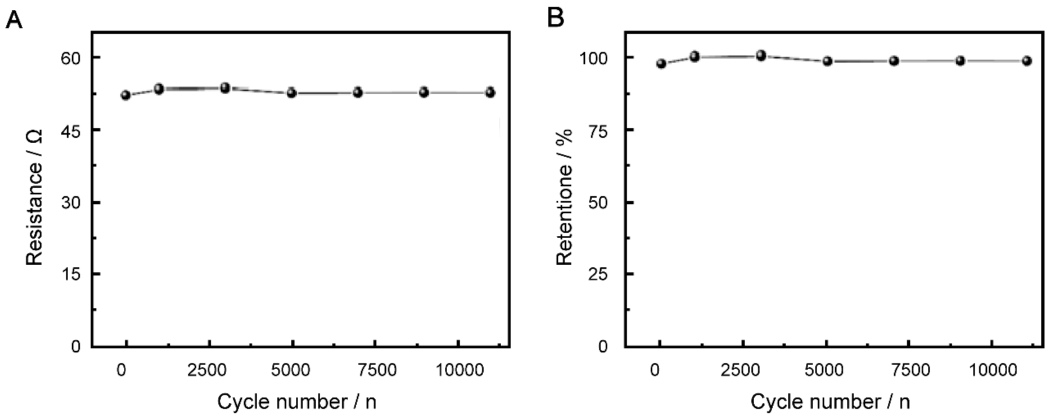

Figure S2. (**A**) The resistance of current collector after stretching at 250%. (**B**) The change of the current collector resistances at 250% after stretching.

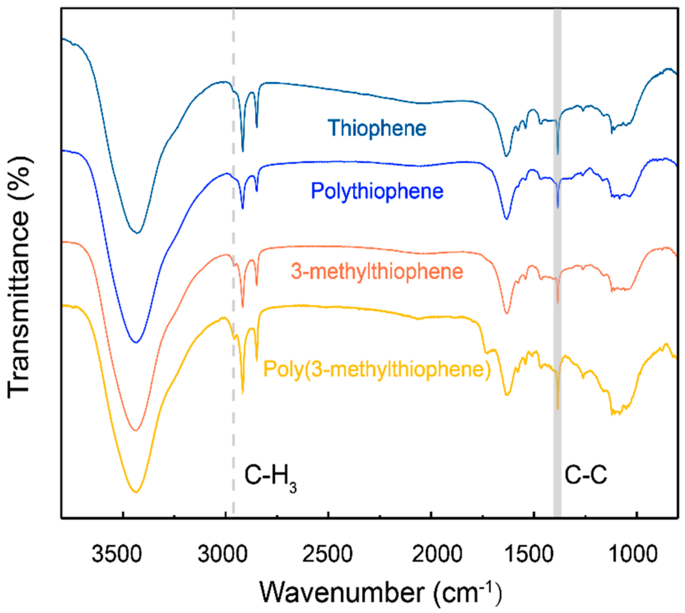

Figure S3. FTIR spectra of thiophene, polythiophene, 3-methylthiophene, and poly(3-methylthiophene).

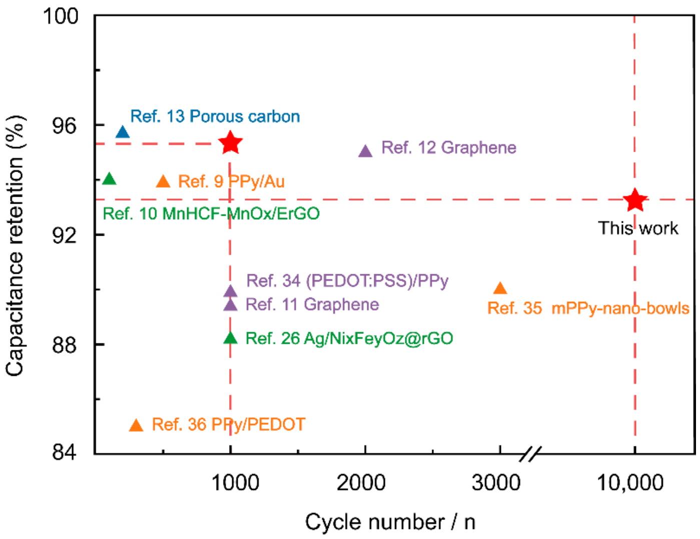

Figure S4. Comparison of conducting polymer electrodes protected by gel electrolyte with previously reported electrode materials under varying cyclic deformation duration.

9. Chen, S.; Shi, B.; He, W.; Wu, X.; Zhang, X.; Zhu, Y.; He, S.; Peng, H.; Jiang, Y.; Gao, X.; Fan, Z.; Zhou, G.; Liu, J.; Kempa, K.; Gao, J. Quasifractal Networks as Current Collectors for Transparent Flexible Supercapacitors. Adv. Funct. Mater. 2019, 48, 1906618.
10. Liang, J.; Tian, B.; Li, S.; Jiang, C.; Wu, W. All-printed MnHCF-MnOx-based high-performance flexible supercapacitors. Adv. Energy Mater. 2020, 12, 2000022.
11. Shao, Y.; Li, J.; Li, Y.; Wang, H.; Zhang, Q.; Kaner, R. Flexible quasi-solid-state planar micro-supercapacitor based on cellular graphene films. Mater. Horizons. 2017, 6, 1145-1150.
12. Choi, B.; Chang, S.; Kang, H.; Park, C.; Kim, H.; Hong, W.; Lee, S.; Huh, Y. Huh, High performance of a solid-state flexible asymmetric supercapacitor based on graphene films. Nanoscale. 2012, 416, 4983-4988.
13. Cao, M.; Feng, Y.; Tian, R.; Chen, Q.; Chen, J.; Jia, M.; Yao, J. Free-standing porous carbon foam as the ultralight and flexible supercapacitor electrode, Carbon. 2020, 161, 224-230.
26. Liu, T.; Yan, R.; Huang, H.; Pan, L.; Cao, X.; DeMello, A.; Niederberger, M. A micromolding method for transparent and flexible thin-film supercapacitors and hybrid supercapacitors. Adv. Funct. Mater. 2020, 30, 2004410.

34. Teng, W.; Zhou, Q.; Wang, X.; Che, H.; Hu, P.; Li, H.; Wang, J. Hierarchically interconnected conducting polymer hybrid fiber with high specific capacitance for flexible fiber-shaped supercapacitor. Chem. Eng. J. 2020, 390, 124569.
35. Cui, J.; Xing, F.; Luo, H.; Qin, J.; Li, Y.; Zhong, Y.; Wei, F.; Fu, J.; Jing, C.; Cheng, J.; et al. General synthesis of hollow mesopo-rous conducting polymers by dual-colloid interface co-assembly for high-energy-density micro-supercapacitors. J. Energy Chem. 2021, 62, 145–152.
36. Diao, Y.; Woon, R.; Yang, H.; Chow, A.; Wang, H.; Lu, Y.; D'Arcy, J.M. Kirigami electrodes of conducting polymer nano-fibers for wearable humidity dosimeters and stretchable supercapacitors. J. Mater. Chem. A 2021, 9, 9849–9857.
